# Information and Communication Technologies in the Care of the Elderly: Systematic Review of Applications Aimed at Patients With Dementia and Caregivers

**DOI:** 10.2196/rehab.5226

**Published:** 2016-05-02

**Authors:** Claudia I Martínez-Alcalá, Patricia Pliego-Pastrana, Alejandra Rosales-Lagarde, JS Lopez-Noguerola, Eva M Molina-Trinidad

**Affiliations:** ^1^Consejo Nacional de Ciencia y TecnologíaResearch FellowCiudad de MexicoMexico; ^2^School of Health SciencesDepartment of GerontologyUniversidad Autónoma del Estado de HidalgoPachucaMexico; ^3^Consejo Nacional de Ciencia y TecnologíaResearch FellowCiudad de MéxicoMexico; ^4^Division of Molecular PsychiatryDepartment of Psychiatry and PsychotherapyUniversity Medicine GöttingenGöttingenGermany; ^5^School of Health SciencesDepartment of MedicineUniversidad Autónoma del Estado de HidalgoPachucaMexico

**Keywords:** information and communication technologies, eHealth, elderly, caregiver, Alzheimer's disease, systematic review

## Abstract

**Background:**

The interest in applying information and communications technology (ICT) in older adult health care is frequently promoted by the increasing and unsustainable costs of health care services. In turn, the unprecedented growth of the elderly population around the globe has urged institutions, companies, industries, and governments to respond to older adults’ medical needs.

**Objective:**

The aim of this review is to systematically identify the opportunities that ICT offers to health services, specifically for patients with dementia and their families.

**Methods:**

A systematic review of the literature about ICT applications that have been developed to assist patients with Alzheimer’s disease (AD) and their primary caregivers was conducted. The bibliographic search included works published between January 2005 and July 2015 in the databases Springer Link, Scopus, and Google Scholar. Of the published papers, 902 were obtained in the initial search, of which 214 were potentially relevant. Included studies fulfilled the following inclusion criteria: (1) studies carried out between the years of 2005 and 2015, (2) studies were published in English or Spanish, (3) studies with titles containing the keywords, (4) studies with abstracts containing information on ICT applications and AD, and (5) studies published in indexed journals, proceedings, and book chapters.

**Results:**

A total of 26 studies satisfied the inclusion criteria for the current review. Among them, 16 were aimed at the patient with AD and 10 at the primary caregivers and/or family members. The studies targeted applications that included assistive technology (44%, 7/16), telecare (37%, 6/16), and telemedicine (31%, 5/16). The information systems (56%, 9/16) and Internet (44%, 7/16) were the most commonly used enabling technologies for the studies. Finally, areas of attention more covered by the studies were care (56%, 9/16), treatment (56%, 9/16), and management (50%, 8/16). Furthermore, it was found that 20 studies (77%, 8/26) evaluated their ICT applications through carrying out tests with patients with dementia and caregivers.

**Conclusions:**

The key finding of this systematic review revealed that the use of ICT tools can be strongly recommended to be used as a lifestyle in the elderly in order to improve the quality of life for the elderly and their primary caregivers. Since patients with AD are completely dependent in most activities, it is necessary to give attention to their primary caregivers to avoid stress and depression. In addition, the use of ICT in the daily life of caregivers can help them understand the disease process and manage situations in a way that is beneficial for both parties. It is expected that future developments concerning technological projects can support this group of people.

## Introduction

New information and communication technologies (ICT), including Internet and mobile technology, have become essential tools in most sectors of modern societies, including the health care sector. While et al have defined ICT as instruments and procedures that allow the acquisition, production, treatment, communication, registry, and presentation of information in the form of voice, images, and data contained in acoustic, optical or electromagnetic signals [1]. In an increasingly aging society, it is necessary to establish new alternatives that attempt to satisfy the needs of the elderly and that, at the same time, improve their quality of life. In this context, the term electronic health (eHealth) is widely used by many people, institutions, professional organizations, and financial entities to refer to the adoption of ICT in the field of health care. Meier et al mention that eHealth is an emerging field that bridges health informatics, public health, and the private sector, and it refers to health services and the information that is delivered or improved through Internet and related technologies [2]. For their part, the World Health Organization (WHO) and the Pan American Health Organization (PAHO) define eHealth with regard to the support provided by the cost-effective and safe use of ICT in health and related areas, including health care services, vigilance, and documentation, as well as education, knowledge, and research on health issues [3,4]. It is also important to point out that eHealth offers a set of advantages, for instance (1) supports information exchange; (2) improves access to health care; (3) reduces costs; and (4) improves public and individual health through personalized medicine [3,5,6].

The attention provided by ICT applications in the field of medical care offers plenty of benefits for the elderly. Several studies indicate that the most widely used technologies in medical services are telemedicine and teleassistance [7,8], and recently, mobile assistance is gaining more popularity [9]. For example, the work of Cash et al discussed the use and application of assistive technology for people with dementia, taking into account ethical considerations. In this study, "assistive technologies for smart homes, telecare, and low-level technology" are recognized as accessible tools in the public market [10]. On the other hand, Lauriks et al proposed the use of ICT and Global Positioning System (GPS) technology to assist informal caregivers of people with dementia in their care giving role [11]. Likewise, Evans et al performed a systematic review to investigate how assistive technologies are being designed to help patients with dementia and their caregivers. They found that the use of assistive technologies focuses on the support of daily activities, safety monitoring, memory aids, and preventing social isolation, improving the ease of living, and also that many elderly individuals prefer to stay at home when aging. Thus, there is an urge to remain an independent and functional person during the old age and assistive technologies could help to achieve this [12].

However, ICT applications used in health services present some limitations. One of them is that older adults are frequently resistant to the use of new technologies, in particular to the acquisition of new knowledge and skills necessary for the use of electronic devices and information systems. On the other hand, Alzheimer’s disease (AD) is the main cause of dementia among older adults. Dubois et al indicate that AD is clinically expressed as a slowly progressive dementia that tends to have insidious onset and that generally starts with recent memory failure and ends with a completely dependent, bedridden patient [13]. Providing care to a patient with AD can bring high physical, emotional, and financial costs to the patient’s family, the health care institution, and the government. Likewise, overload of the caregiver due to the demands of the patients with AD, even more if the caregiver is an older adult herself, can have negative repercussions on the caregiver’s quality of life, mainly due to psychiatric problems like anxiety and depression [14].

From a technological perspective, both the patient and the caregiver are more willing to assume a proactive role in the use of ICT with the purpose of carrying out in-home diagnoses and treatment to improve quality of life. In this context, the caregivers require technological tools that enable them to provide better care as well as timely and efficient attention to the partially or totally dependent patient. These trends have contributed to the strong conviction that ICT can offer useful and efficient tools that improve elderly patients with AD quality of life and, at the same time, provide a support for their family and/or caregivers. Applications and services that are currently being developed seek to facilitate quality enhancement, equality, and access to social and medical care [11].

The purpose of this article is to integrate the knowledge we have about older adults with AD and the opportunities that ICT offers to health services, specifically for this group of patients and their families. For that purpose, this article is divided in six sections. The first section introduces the problem and provides some basic definitions that will be used throughout the study. In the second section, AD as well as its relationship with aging is defined. The third section focuses on caregivers, since these persons have a very close relationship with patients with AD. In the fourth section, the most relevant ICT applications for AD patients and their caregivers are analyzed and classified according to the typology of application, technology, and their domain of application. The innovation opportunities provided by ICT in the field of health care are presented in the fifth section. Finally, conclusions and further work are discussed in the last section.

### Alzheimer’s Disease and its Relationship With Aging

Due to the increasingly aging population, AD has become a problem of great medical and social repercussions. Dementia is a chronic and progressive syndrome characterized by the deterioration of cognitive and behavioral functions causing disability, dependency, and low self-esteem. AD is the most common type of dementia, representing between 60-80% of dementia cases [15]. AD is defined as a progressive neurodegenerative disease characterized by a series of clinical and pathological features of relative variability [16].

**Figure 1:**
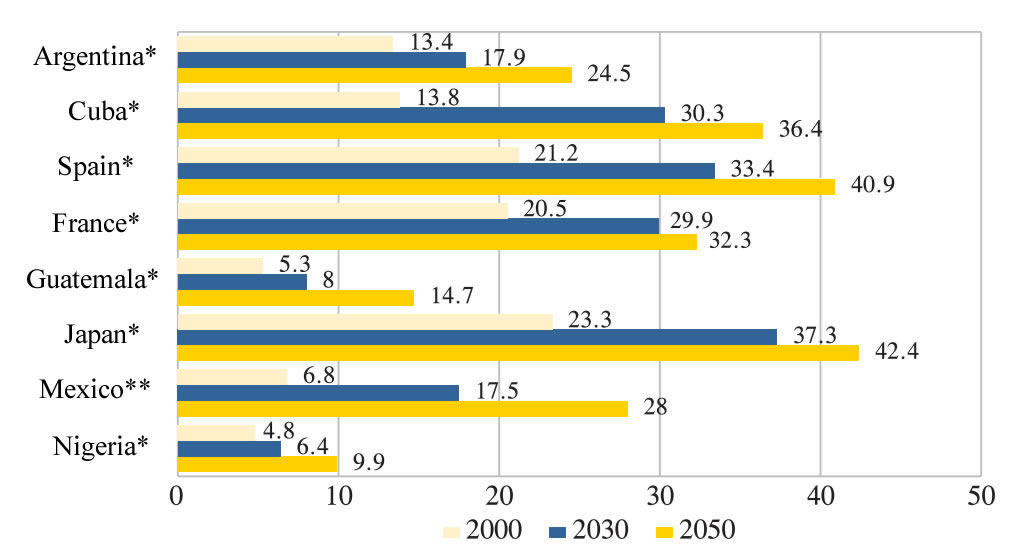
Percentage of the population aged 60 years and older in the years 2000, 2003, and 2050. Sources include the World Population Prospects, the 2002 revision and CONAPO, Proyecciones de la Población 2010-2050.

Considering the foreseen tendencies of global demographic aging, it is estimated that by 2050 there will be around 2 billion older adults (Figure 1). In 2010, there was an estimated 35.6 million people with dementia around the world and it is expected that this figure will duplicate every 20 years, reaching 65.7 million and 115.4 million in 2030 and 2050, respectively [17]. The increase of dementia will be more dramatic in low and middle income countries where more than two thirds of the number of cases will be diagnosed in 2050 [18]. In the following twenty years, it is expected that the number of people diagnosed with dementia will increase 40% in Europe, 63% in North America, 77% in the Latin America (eg, Mexico and Argentina), and 89% in developed countries of Asia-Pacific [18].

This situation is an important public health challenge, since it represents one of the major health care and social problems faced by public health at a global scale. For this reason, health professionals and researchers have set out to look for alternative solutions to provide support to this vulnerable group [18]. Similarly, associations related to AD are willing to support their governments with timely data and information to design action plans that ensure high-quality assistance and support for people with dementia and their caregivers.

In order to measure cognitive decline in older adults several trial tests have been designed. One of them is the Mini-Mental State Examination (MMSE) [19]. This test consists of five different sections that include a series of questions and problems related to space-time orientation, information registration, attention, algebraic operations, recall and repetition of short phrases, delayed memory recall, identification of everyday objects, verbal comprehension, writing abilities and medium-complexity drawing abilities [19,20]. MMSE *,* also called Folstein’s Test, was modified for its application in different countries (eg, English and Spanish versions). Older adults living in rural areas are more likely to develop cognitive decline, which is presumed to be caused by the low educational level [21].

### The Primary Caregiver and her Relationship With Alzheimer’s Disease

Patients with AD, especially those with mild and moderate levels of dementia, receive most care in their homes from a family member. This event is more common in developing countries, like Mexico, due to poor coverage of health and social care systems.

The primary caregiver is the person who attends to the physical and emotional needs of a person who is ill or disabled. This role is generally assumed by the husband and/or wife, son and/or daughter, a relative or a person who is close to the patient. The work of a primary caregiver gains more relevance for the group that surrounds the patient as the illness progresses, not only because of the direct attention the patient requires, but also for their role in the reorganization, maintenance, and cohesion of the family. Likewise, the primary caregiver assumes total responsibility for the patients, assisting them in the execution of all the activities that they are unable to perform. Usually the caregiver does not receive economic support or patient care training [22].

Family members and caregivers are the main support for any patient. According to the literature, it has been observed that a median average of 1.6 hours a day are invested in providing care to patients with AD and with other types of dementia, and assisting them in activities of daily living such as bathing, dressing, grooming and eating. Similarly, 3.7 hours a day are spent in assisting them with instrumental activities of daily living such as cooking, shopping, and domestic economies, and 7.4 hours a day are invested in general supervision [23]. All this can significantly affect the primary caregiver by causing stress, work overload, depression, as well as physical and medical problems. Behavioral alterations common among people with dementia tend to be misunderstood causing stigma, guilt, and discomfort in caregivers.

The work overload endured by primary caregivers, once the available resources are exhausted, can have a negative effect on their health. Numerous works on these negative repercussions have been published, where references to psychiatric malaise are abundant (mainly anxiety and depression) [24]. As well, other important repercussions have been described such as negative effects on the caregiver’s physical health [25], social isolation [26], lack of free-time [27], poor quality of life or deterioration of economic situation [28]. All of these factors might contribute to what some authors call the caregiver syndrome [14,29].

Nowadays, family members, caregivers, and health professionals show a constant demand of technologically supported tools. Some of the most demanded technologies are systems that improve diagnosis and/or treatment of certain illnesses, and systems that improve communication with the patient or that facilitate assistance and remote monitoring of the patient with the use of different health and social care resources. In this context, it is important that family members and caregivers receive social support (ie, personal interaction, feedback, information, and training), that allows them to understand their role as caregivers [30]. This can be achieved by the adoption of easy-access ICT tools. ICT can provide support to family members and the caregiver and can even allow access to social environments (eg, social networks), all of which brings more autonomy, quality of life, and social inclusion to both the patient and the caregiver [31].

## Methods

### Procedure

In order to gather more knowledge on ICT applications that have been developed to assist patients with AD and their primary caregivers, a literature review was carried out by two of the main authors of this paper (CIMA and PPP). While the review was performed independently, the authors, maintained constant communication to ensure common agreement. The literature review was performed using a systematic review protocol. Systematic review is a method to evaluate and interpret relevant research works available for analysis dealing with a topic or phenomenon of interest [32]. At the first stage of the literature review process, the following research questions were formulated: (1) What modalities are currently being used for the development of ICT applications? (2) What technologies are most widely used at the moment of developing an ICT application? and (3) What domains of application are mainly covered by ICT applications? These questions allowed for the identification of the modalities used and the technologies applied for the development of such applications. In addition, they allowed us to classify the areas of attention that are contemplated in the development of such applications. In order to respond to each of the research questions, the key words ICT applications, AD, dementias, older adult, elderly, aging, and caregiver were identified. The search strings generated in order to obtain published works were ICT applications and AD, ICT applications and older adult, and ICT applications and caregiver and AD.

### Inclusion and Exclusion Criteria

The inclusion criteria for the examined studies were (1) carried out between the years of 2005 and 2015; (2) in English or Spanish; (3) titles containing the keywords; (4) abstracts containing information on ICT applications and AD; and (5) published in indexed journals, proceedings, and book chapters. Studies that were not published during the indicated period and that did not include relevant information on ICTs applications were excluded.

## Results

Each study was analyzed taking into account the modalities, technologies, and areas of attention that were considered in each ICT application developed. This was done with the purpose of identifying which area is given more coverage and which needs to be given more attention. The following sections detail the modalities, technologies, and areas of attention identified in the systematic review.

### Modalities Employed for the Development of ICT Applications

The description of the modalities considered in the development of an ICT application focused on health care are outlined in Textbox 1.

**Modalities considered in the development of an ICT application.**

Modality

- Telemedicine: Use of ICT to provide remote medical service (eg, teleconsultation, telehealth, telegerontology, telemonitoring, telerehabilitation, teletherapy, and tele-education)
- Teleassistance: Use of ICT to offer remote social and health care assistance to patients in their homes (ie, basic teleassistance, video-assistance, and telealarm
- Assistive technologies: Use of technologies to provide support to individuals with a disability or with special needs (ie, ambient assisted living (AAL), assistive technologies, virtual assistance, and domotics)
- Communication: Use of means of communication to assist and/or support individuals with a disability or with limitations, and their relatives (ie, telephony, radio, email, television, satellites)
- Location: Use of means of location for the transmission of real-time location (ie, GPS and global navigation satellite system (GNSS)
- Electronic services (e-services): Use of ICT to access information and digital content (e-services, digital contents, electronic assistance (e-assistance))
- Mobile health (mHealth): Use of mobile technology for medicine and public health practices. These applications allow data collection, medical information delivery, real-time monitoring of patients’ vital signs, and direct health care provision.

### Technologies for Electronic Health

In this section, enabling technologies that are most frequently applied for the design of eHealth applications were identified (Textbox 2).

**Technologies enabled for the design of eHealth applications.**

Technology type and subtype

- Internet
  - e-services, blogs, digital contents, Web 2.0, social networks, collaborative platforms
- Information systems
  - User interface, touch screens, virtual agents
- Telecommunications
  - Mobile phones, video conference, digital terrestrial television (DTT), television, satellites
- Ambient intelligence
  - Ambient intelligence systems, sensors, sensor networks, wireless sensor networks, domotics
- Signal processing
  - Image processing, video processing, signal analysis, pattern recognition, 3D images
- Robotics
  - Robotic assistants
- Virtual reality
  - Simulations and stimulation through consoles
- Geolocation
  - GPS

### Areas of Attention

In order to provide a wider classification of ICT applications developed for patients with AD and their caregivers, the areas of attention identified are shown in Textbox 3.

**Areas of attention identified in the systematic review.**

Area of attention

1. Research (discover causes): the need to increase knowledge in order to understand the cause(s) of the disease and to provide products that meet the needs of the patient.
2. Treatment (therapy): support for individuals with AD and maximization of their capabilities through safe and effective interventions so that they can act more independently.
3. Diagnosis: timely diagnosis of AD through cognitive and brain activation exercises.
4. Care: provision of medical and social care through the interchange of improved practices in care for patients with AD and their self-care induction.
5. Prevention: action plans for AD through early intervention and epidemiologic studies.
6. Quality of life: psychological support to the patient, family members, and caregivers and advice and promotion of elderly well-being.
7. Awareness raising and social mobilization: promotion of solidarity, mobilization and social engagement, and sensitization of public opinion.
8. Management (monitoring): promotion of assisting services for patients and primary caregivers.

### Included Studies

The bibliographic search carried out for this study included works published between January 2005 and July 2015. The searched databases were Springer Link, Scopus, and Google Scholar. A total of 902 results were obtained from the three selected information sources. The Springer Link search was limited to journal articles and it returned 371 references whereas the Scopus database included conferences and journal articles and yielded 19 results. Finally, Google Scholar included congress and journal articles, and book chapters, and returned 522 references. The databases selected in this review were set to maximize the search results of studies in Spanish.

**Figure 2:**
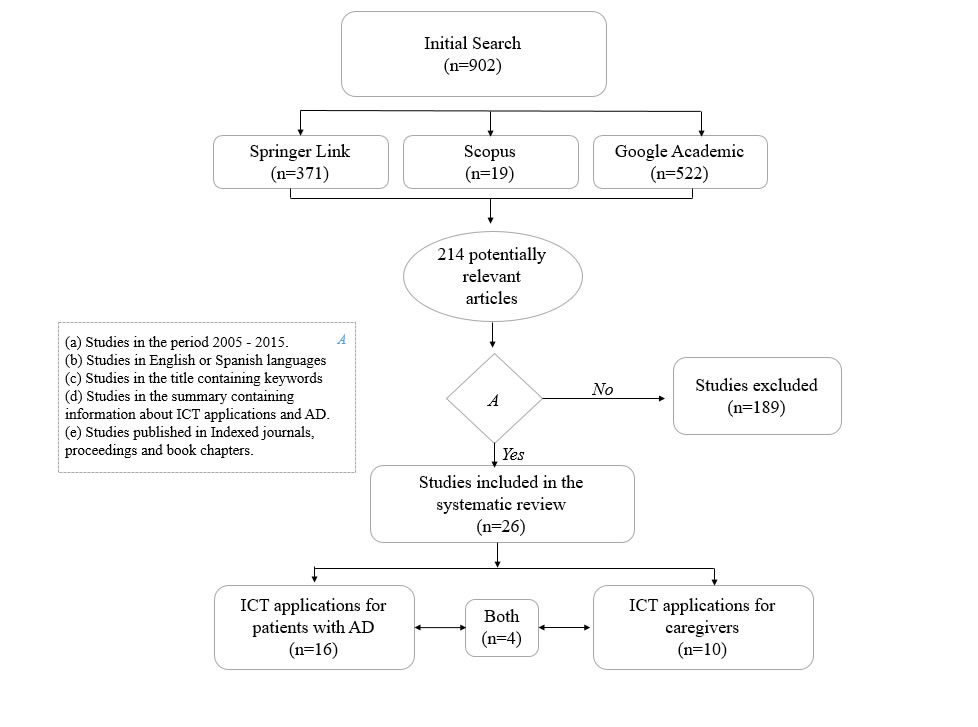
The systematic review carried out in this study.

Abstracts and titles were sufficient to rule out papers that did not meet inclusion criteria (points 3 and 4). When it was not clear from the abstracts and titles whether they included ICT (inclusion criteria 4), the full paper was reviewed. References in the selected articles and previous published reviews were also analyzed with the aim of identifying additional studies that had not been identified through the searched databases. Using this method, 4 Springer Link papers, 14 Scopus papers, and 8 Google Scholar papers were detected. In total, 26 studies were obtained where 16 studies described ICT applications aimed at patients with AD and other dementias, and the other 10 described ICT applications aimed at caregivers and/or family members. It is noteworthy that 4 of the 26 studies were directed to both cases (Figure 2).

The results obtained from the systematic review are categorized into the following two groups: (1) ICT applications aimed at patients with AD and other dementias (Multimedia Appendix 1) [33-48], and (2) ICT applications aimed at the primary caregiver (Multimedia Appendix 2) [49-58]. It is important to point out that in each study several areas of attention and enabling technologies were included.

### Research Outcomes

**Figure 3:**
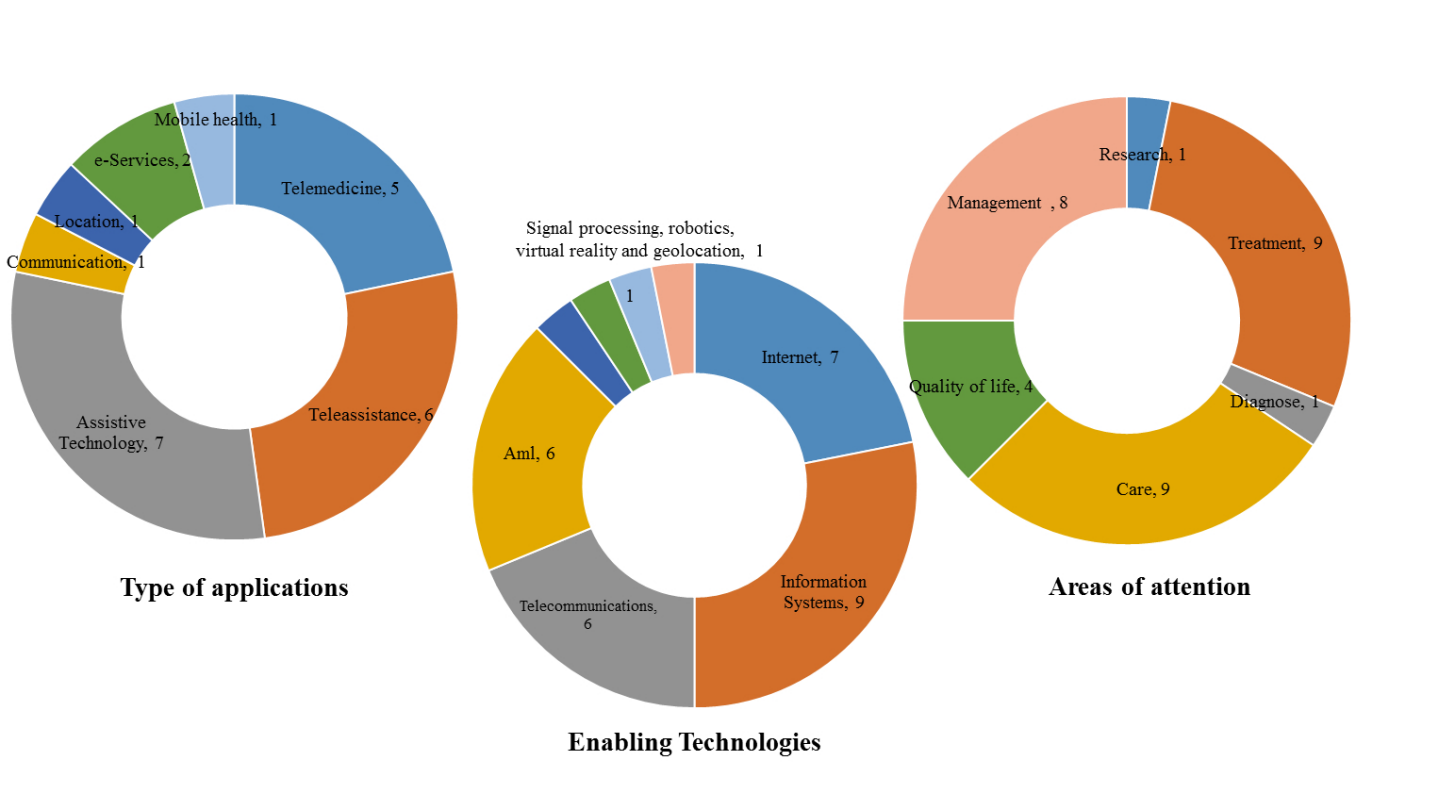
Analysis of ICT applications used by patients with Alzheimer's disease and other dementias.

ICT applications used for patients with AD and other dementias were analyzed and the results are shown in Figure 3 and Multimedia Appendix 1. The most commonly used application types are assistive technology (44%, 7/16) and teleassistance (37%, 6/16). The most commonly used enabling technologies are information systems (56%, 9/16) and Internet (44%, 7/16). Signal processing (6%, 1/16), robotics (6%, 1/16), virtual reality (6%, 1/16), and geolocation (6%, 1/16) are less frequently used. The areas of attention most frequently covered are care (56%, 9/16), treatment (56%, 9/16), and management (50%, 8/16). The areas of attention less frequently addressed are research (6%, 1/16) and diagnosis (6%, 1/16). It is important to point out that the studies indicated in references [38,39] are projects previously developed in Mexico. These studies are important because there is little evidence of these types of projects in Mexico, to our knowledge.

**Figure 4:**
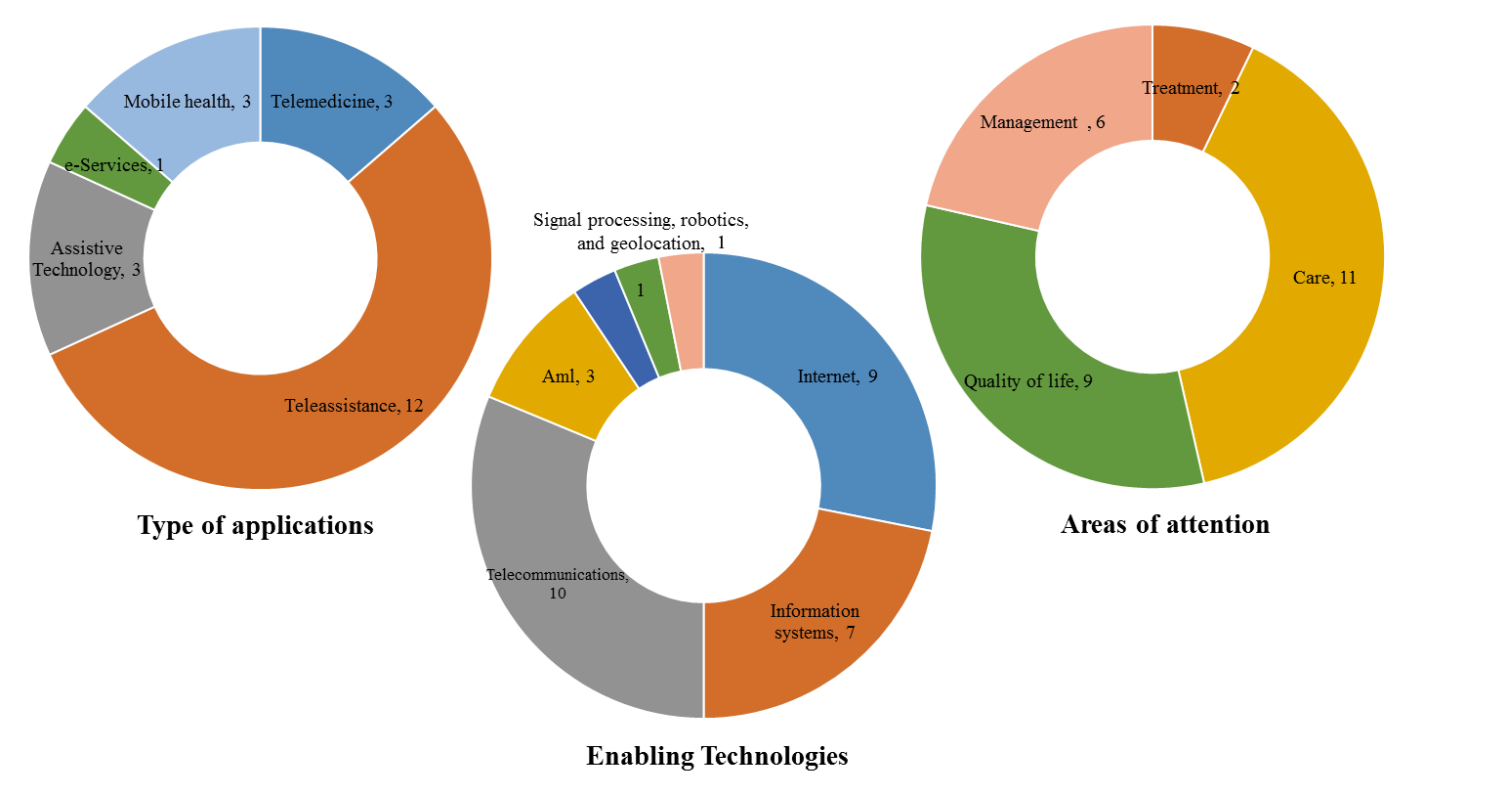
Analysis of ICT applications used by the primary caregiver.

ICT applications used by primary caregivers of patients with dementia were also analyzed (Figure 4,Multimedia Appendices 1 and 2). The most commonly used applications by primary caregivers are teleassistance applications (86%, 12/14). Less frequently used applications are mobile health (21%, 3/14), assistive technology (21%, 3/14), and telemedicine (21%, 3/14). The most commonly used enabling technologies are telecommunications (71%, 10/14), Internet (64%, 9/14), and information systems (50%, 7/14), whereas ambient intelligence (21%, 3/14), signal processing (7%, 1/14), robotics (7%, 1/14), and geolocation (7%, 1/14) are less frequently used. Finally, the most covered areas of attention are care (79%, 11/14) and quality of life (64%, 9/14), whereas management (43%, 6/14) is less frequently used. It should be mentioned that the studies [37-39,42] were taken into consideration in the analysis of ICT applications used by the caregiver.

## Discussion

### Principal Findings

The aim of this review is to systematically identify the opportunities that ICT offers to health services, specifically for patients with dementia and their families.

Here, we identified 26 studies, among which 16 presented projects aimed at patients with AD and 10 at the primary caregiver. From these 16 studies, 9 focused on patients with AD (56%, 9/16), 5 (31%, 5/16) on patients diagnosed with mild cognitive impairment according to the (MMSE), and only one study was focused on patients with mild dementia. Furthermore, it was found that 13 studies (81%, 13/16) evaluated their ICT applications through carrying out tests with patients.

Among the studies that used assistive technologies, study [33] was found to have carried out 46 tests with patients with mild cognitive impairment, who were suspected to have AD and who were under treatment (cholinesterase inhibitors). Results indicated that after 12 weeks of using interactive multimedia Internet-based system (IMIS System), along with the Integrated Psycho-Stimulation Program (IPP), patients had improved their initial scores in the Alzheimer's Disease Assessment Scale-Cognitive subscale (**ADAS** - **Cog**) and MMSE tests, maintaining their scores during 24 follow-up weeks. Similarly, in study [35], 8 patients with mild dementia remained stable after using a televideo monitoring system *,* while the control group, who did not use the system, showed less stability, indicating a significant difference between the two groups.

Robert et al presented the Sweet Home ANR project, which aims to help patients with mild cognitive impairment perform daily living activities [41]. The tests carried out with patients took place in rooms equipped with audio and video sensors. For the language activities, the system was tested with 21 healthy patients and 24 patients with AD, while the walking test was performed with 17 healthy patients and 16 patients with AD. In the study, they showed that Sweet Home ANR was capable of detecting the full set of activities carried out by the patients. Likewise, the system enabled the differentiation of patients with AD from healthy patients using the video monitoring system (VMS) functional score. Casacci et al [47] presented the ALTRUISM project *,* in which tests were carried out with 20 patients with AD with the aim of performing remote supervision, through a virtual personal trainer, of rehabilitation exercises and routines executed by these patients. The results showed important and promising data concerning the use of ICT systems of remote rehabilitation.

Among the studies that used teleassistance applications, we found one written by Garcia et al [36] that presented a multi-agent ambient intelligence system aimed at improving attention to and health care of patients with AD living in geriatric residential settings. Although this study did not present an evaluation of the application through tests with patients, it made a comparison with a former version of the system and confirmed that ALZ-MAS 2.0 is much more robust and has better performance than the older version.

Of the 14 studies presenting projects aimed at the primary caregiver, including those shown in Multimedia Appendix 2 , it was found that 11 studies focused on informal caregivers (family members), and 7 focused on formal ones (health professionals). Likewise, it was found that 10 of the 14 studies evaluated their ICT applications through tests with caregivers.

As previously shown, teleassistance is the most commonly used application for caregiver support. In this context, study [50] evaluated the effectiveness of the eCare system with family members older than 21 years old who lived or were in the same geographic area as the patient with dementia. The intervention was carried out over a span of approximately 6 months and the results showed a significant decrease in the family member’s workload after using the eCare system. Similarly, it was found that caregivers who presented depression signs at the onset of the intervention significantly improved their state after the intervention. Another study that showed a decrease in stress signs in formal caregivers of patients with dementia is the one carried out by Sugihara et al [53], where 16 formal caregivers were interviewed in order to know the benefits of using the Support Environment system.

Skorupska et al presented the design and implementation of a multimedia platform called Understaid, which provides support to the family of patients with dementia [57]. This platform was evaluated by 40 caregivers attending patients with different levels of dementia. Caregivers provided information of daily care activities, as well as the patient’s behavior through the platform. Although results obtained from the evaluations are not mentioned in this study, the system promises to be a useful tool for caregivers. Another study that was validated by caregivers is the one carried out by Bourennane et al [42], where they describe the experiments performed with patients with AD in a hospital in France. The homecare monitoring system was tested with an 84 year resident and the follow-up was performed by hospital personnel. Results showed that this system is functional and that it can be used for other cases. However, two validations are needed: (1) patients and their family members’ consent, and (2) doctors’ interest and the necessary arrangements for the system connection.

Among the studies using mobile technology for the assistance of primary caregivers, study [56] carried out semi-structured interviews with 9 informal and 2 formal caregivers in its Phase 1. In Phase 2, 2 patients and 4 caregivers were interviewed in order to evaluate low-fidelity prototypes of the mobileWAY application, and in Phase 3, 5 patients and 10 caregivers were interviewed to evaluate interaction and usability of the application. Finally, caregivers were invited to answer usability evaluations one more time. These evaluations showed promising results, not only in terms of general comprehension of the mobileWAY system, but also in terms of ease of use and potential interest in using the application in the future.

The present review demonstrates that ICT has great potential for supporting the health care field. As we were particularly interested in the study of AD and other dementias that are affecting the aging population, we identified different innovation opportunities that can be designed in order to enhance the quality of life of older adults with dementia and their family members. These innovation opportunities are (1) information services for health professionals which facilitate information exchange and enable access to knowledge on a variety of treatments and practices; (2) more complete information services for counseling and education of patients and informal caregivers; (3) information services for diagnosis, treatment and/or rehabilitation of patients; (4) virtual communities providing psychological support that also allow contact with health professionals; and (5) regarding technology acceptance, the design of strategies for the older adult to integrate the use of ICT tools in his/her daily life should be considered.

### Limitations

One limitation of the review is that studies were searched in three databases (Springer Link, Scopus, and Google Scholar) using specific search strings, so it is possible that this searching strategy did not identify some eligible studies. In order to tackle this, manual searches were performed in previous reviews and key journals. Despite the exhaustive search, additional eligible studies were not identified using this method. Another possible limitation of this systematic review is that only documents written in English and Spanish were considered.

### Conclusions and Further Work

According to the results obtained from the systematic review, most ICT applications developed for both patients with any type of dementia and primary caregivers are focused on in-home care (teleassistance). The use of ICT in older adults with dementia can be implemented as a lifestyle in order to improve the quality of life of the elderly and their primary caregivers. Given that AD is a degenerative disease which causes progressive deterioration of memory, their primary caregiver’s quality of life can be undermined as the disease becomes increasingly severe, which can cause depression in the primary caregiver. In addition, the use of ICT in the daily life of caregivers can help them understand the disease process and manage situations in a way that is beneficial for both parties. As a consequence, this can enhance interpersonal relationships and promote social harmony for both the elderly and their caregiver. Furthermore, the systematic review of ICT applications for patients with AD and other dementias reflected low presence of innovative projects in less developed countries, such as Mexico. Future work is being developed on the design of strategies for older adults to integrate the use of ICT tools in their daily life. With this, progress can be made in the development of technological projects that support this group of people.

## Multimedia Appendix 1

ICT applications aimed at patients with AD and other dementias.

## Multimedia Appendix 2

ICT applications aimed at the primary caregiver.

## Abbreviations

AD: Alzheimer´s disease
eHealth: electronic health
e-service: electronic service
GPS: Global Positioning System
ICT: information and communication technologies
MMSE: Mini-Mental State Examination
