# Supplementary material for: Information and Communication Technologies in the Care of the Elderly: Systematic Review of Applications Aimed at Patients With Dementia and Caregivers
Source: JMIR Rehabil Assist Technol. 2016 May 2;3(1):e6. doi: 10.2196/rehab.5226 (PMC5454565; doi:10.2196/rehab.5226)
Supplement: Supplementary file 1 [file rehab_v3i1e6_app1.pdf]

English Languages Studies

**Year**	**Name of application**	***Database***	***Objective***	***Type of patient***	***Type of application***	***Technology***	***Domain of application***	***Significant Findings***
2006	The IMIS [33]	Google Scholar	The IMIS is an interactive multimedia internet-based system for the cognitive stimulation of Alzheimer's disease.	Older adult with AD	Assistive technology	IS<br>Internet	Treatment	This study shows that the IMIS program provided an improvement above and beyond that seen with classic cognitive stimulation, with improvement lasting 24 weeks.Thus, it seems that an individually constrained cognitive stimulation program such as the IMIS used here is more efficacious than treatment only with drugs, and at least augments traditional psychostimulation.
2007	Cogknow [34]	Google Scholar	COGKNOW aims at providing assistance to patients with dementia, offering support in four aspects: remembering things, maintaining social contact, performing activities of daily living and increasing safety.	Older adult with MD	Teleassistance<br>Assistive Technologies<br>Location	Internet<br>IS<br>Geolocation<br>AmI	Treatment<br>Quality of life	The Cogknow project helps people with mild dementia to navigate through their day and imporve their independence and quality of life. However, the Cogknow it is still a prototype.
2007	Televideo monitoring [35]	Scopus	Televideo monitoring aims at monitoring medication compliance of patients with mild dementia	Patients with MD	Telemedicine	IS<br>Telecomunications	Management<br>Care	This study provides encourag- ing results for the ability of a home telehealth appli- cation to positively affect mild dementia patients.
2008	ALZ-MAS 2.0 [36]	Scopus	ALZ-MAS 2.0 is an Ambient Intelligence based multi-agent system aimed at enhancing the assistance and health care for Alzheimer patients living in geriatric	Persons with AD	Teleassistance	AmI	Management<br>Care	In this study it is demonstrated that the previous version of ALZ-MAS is far more unstable than ALZ-MAS 2.0. Results also show that FUSION@ is adequate for building

English Languages Studies

**Year**	**Name of application**	***Database***	***Objective***	***Type of patient***	***Type of application***	***Technology***	***Domain of application***	***Significant Findings***
			residences.					complex systems and exploiting composite services, in this case ALZ-MAS 2.0.
2009	ALADDIN [37]	Scopus	The ALADDIN aims at supporting maintaining health and functional capability, providing the means for the self-care and the self-management of chronic conditions, providing added value to the individual, leveraging his/her quality of life, while at the same time supporting the moral and mental upgrade of both the patients and their carers.	Elderly with AD<br><br>Caregiver	Teleassistance<br><br>Assistive tecnologies<br><br>e-Services	Internet<br><br>IS<br><br>Aml	Management<br><br>Care<br><br>Quality of life	This study mentions that ALADDIN aspires to have an immediate impact on the quality of life of patients suffering from dementia and their carers.
2009	Intergenerational Communication Systems [38]	Scopus	This system consists of multimodal interfaces and distributed software components that enable the members of the elders' family to motivate them to carry out their Cognitive Stimulation activities.	Older adults with MCI<br><br>Caregiver	Teleassistance	IS<br><br>Internet	Quality of life<br><br>Treatment	This work identified a set of design insights which allowed to integrate the members of the elder's social family network as informal caregivers in cognitive stimulation activities, as well as to integrate them to act as additional motivators for the participation of elders in executing those activities.
2012	CANoE [39]	Scopus	CANoE is a model for the design of context-aware notifications in critical environments, such as a nursing home. The main feature of this model is that it considers	Older adult with MCI<br><br>Caregiver	Assistive technologies	Telecommunications	Management<br><br>Care	The results of this study provide evidence that caregivers achieved an increased awareness of the situations surrounding the elderly care and perceived the systems as adequate

English Languages Studies

**Year**	**Name of application**	***Database***	***Objective***	***Type of patient***	***Type of application***	***Technology***	***Domain of application***	***Significant Findings***
			three sources of context (the environment, the issuer and the receiver of the notification) for adapting the content, the terms of delivery and the presentation of the notification message.					tools to support their coordination while attending a situation of care.
2012	Video Monitoring System [40]	Scopus	The general objective of this study is to demonstrate that it is possible to use a video vigilance system to obtain quantifiable evaluation of instrumental activities of daily living (IADLs) in patients with AD and MCI.	Normal older adult. Older adult with AD and MCI	Communication	Signal processing<br><br>Telecommunications	Management<br><br>Treatment<br><br>Care	The derived daily activity scenario (DAS) scores proposed in this study may improve the prediction of future dementia, and that can be used as an outcome measurement in clinical trials and lead to earlier therapeutic intervention.
2013	Sweet Home ANR [41]	Google Scholar	The SWEET-HOME project aims at assisting MCI patients to perform activities of daily living (ADLs).	Normal older adult and/or with AD	Assistive technologies	AmI	Care<br><br>Diagnose	The monitoring system is able to detect the full set of activities with a detection rate varying from 96.9% to 100%. Regarding activities of daily living, the monitoring system had an average sensitivity of 90% and an average precision of 83.51%. Also, all the participants who accepted to be assessed using the system, indicated that the assessment was perceived as pleasant (83%).
2013	Homecare Monitoring System [42]	Google Scholar	This system is based on a presence multisensor network deployed in the	Older adult with AD	Telemedicine	Telecommunications	Care	The obtained results show that this system is operational and it can be

**Year**	**Name of application**	***Database***	***Objective***	***Type of patient***	***Type of application***	***Technology***	***Domain of application***	***Significant Findings***
			living environment of the monitored dependent person coupled with a wireless identification system. This system allows the nursing staff monitoring the behaviour through a web application accessed remotely, and also intervention in case of dangerous situations thanks to an alert system.	Professional carer	Teleassistance	Internet<br><br>IS<br><br>Aml	Management<br><br>Treatment	technically deployed. However, it needs two complementary validations on a wider cohort of patients. The first is by the patients themselves and their families and the second is more clinical in order to establish the medical interest and the connection with the care system.
2013	ROBADOM Project [43]	Scopus	The ROBADOM project was devoted to the design of a "robot butler", capable of providing verbal and non-verbal interactions and feedbacks for assisting older adults at home.	Older adult with MCI	Assistive technologies	Robotics	Care<br><br>Quality of life<br><br>Research	In this study, the services, cognitive stimulation, reminder and object localization were positively rated. Although the participants considered an assistive robot as useful, they were not yet ready to adopt it. The expressions of the robot were perceived differently in older and young adults. Thus, a robotic system dedicated to older adults should be tailored to the specific characteristics of this population.
2013	TalkMeHome [44]	Google Scholar	TalkMeHome is a service to guide people with mild dementia home using a GPS-enabled smartphone.	Adult with MD	Mobile health<br><br>Teleassistance	Telecommunication	Care	All participants were guided home satisfactorily, even when conditions were suboptimal. Once the connection was made, the use of a smartphone posed no specific problems for the four participants.Communication was found to be good,

English Languages Studies

**Year**	**Name of application**	***Database***	***Objective***	***Type of patient***	***Type of application***	***Technology***	***Domain of application***	***Significant Findings***
								although guiding someone home proved a demanding task for the care professionals.
2013	ePark [45]	Scopus	The main objective of the e-Park system is the detection of cognitive deterioration of a person with Parkinson's disease. This is achieved through a telemedicine system that allows evaluating patients with a disease scale of PD-CRS by using telemedicine system.	Older adult with AD	Telemedicine<br>e-Services	IS<br>Internet	Treatment<br>Management	This study shows the development of the two telemedicine systems, which were developed in real hospital environments in order to focus on monitoring the rehabilitation of patients with neurological disorders. Also, the users showed a favorable result, asserting that telemedicine systems designed are of easy navigation, and therefore, the patients will be able to use it at home.
2014	RGB-D Sensor-based Platform [46]	Scopus	The RGB-D is a digital platform integrating advanced Natural User Interface technologies for multi-domain Cognitive Rehabilitation.	Persons with AD	Telemedicine	IS<br>Internet<br>AmI	Treatment	The proposed platform in this study, allows both the evaluation of the progress of the dementia and the cognitive stimulation of the end-user in several domains.
2015	The ALTRUISM Project [47]	Springer Link	The ALTRUISM project aims at developing a home rehabilitation system through the implementation of a Virtual Personal Trainer in order to remotely monitor and support patients affected by Alzheimer's disease in performing exercises and rehabilitation programs,	Persons with AD	Assistive technology	Virtual reality<br>Telecommunication	Treatment<br>Management	The collected data show a satisfactory integration between the patient and the ALTRUISM system along with a great level of acceptability of this platform by the end-user, both the patients themselves and the caregivers or medical providers, those who, day by day, take care and assist their

English Languages Studies

Year	Name of application	*Database*	*Objective*	*Type of patient*	*Type of application*	*Technology*	*Domain of application*	*Significant Findings*
			autonomously and directly in their home environment.					patients.
Spanish Languages Studies								
	SOCIABLE [48]	Google Scholar	The SOCIABLE project will pilot a radically new ICT-based approach for integrated support of mental activity, as well as boosting of social interaction for individuals that have been diagnosed with mild dementia.	Older adults with different levels of MCI and AD	Telemedicine	IS	Treatment	The preliminary results of the first group of users indicated that participants without cognitive impairment improved in some cognitive variables (MiniMental, attention, mood). In addition, the level of satisfaction with the use of ICTs was high, considering that most of the sample had no previous experience with the use of ICTs.

Abbreviations: MCI: Mild Cognitive Impairment, MD: Mild Dementia, IS: Information System, Aml: Ambient Intelligence
