# Supplementary material for: Information and Communication Technologies in the Care of the Elderly: Systematic Review of Applications Aimed at Patients With Dementia and Caregivers
Source: JMIR Rehabil Assist Technol. 2016 May 2;3(1):e6. doi: 10.2196/rehab.5226 (PMC5454565; doi:10.2196/rehab.5226)
Supplement: Supplementary file 2 [file rehab_v3i1e6_app2.pdf]

Year	Name of application	*Database*	*Objective*	*Type of group*	*Type of application*	*Technology applied*	*Domain of application*	*Significant Findings*
2005	AlzOnline [49]	Scopus	AlzOnline is a Web and telephone-based education and support network for caregivers of individuals with progressive dementia.	Informal caregivers	Teleassistance	Internet<br>IS<br>Telecommunication	Care<br>Quality of life	AlzOnline has the potential of serving as a prototype for other state elder care agencies and provider networks to follow. However, before AlzOnline and other newly developed Internet-based alternative health care delivery ap proaches are widely adopted, further research is needed to assess their efficacy, cost-effectiveness and genezalizability across different caregiver populations.
2007	eCare [50]	Scopus	eCare is a technology-based psychoeducational intervention for family caregivers of dementia patients.	Informal caregivers	Teleassistance	Telecommunication	Care<br>Quality of life	The findings show that existing technology can be used to deliver a multicomponent intervention effectively and efficiently for the caregivers. Besides, the caregivers were very receptive to using the technology and found it easy to use.
2009	Social Support Online [51]	Springer Link	The applications is an online platform where caregivers of patients suffering from Alzheimer's disease can share social support.	Informal caregivers	Teleassistance	IS<br>Internet	Care<br>Quality of life	In this study, the benefits of designing online systems for social support for the family caregivers are presented. These systems help alleviate distress by providing greater social support to caregivers. However, the platform that presented in this study is not yet evaluated.
2009	iCOPE [52]	Springer Link	iCOPE aims at providing caregivers and family members with a monitoring system for	Caregivers and family members	Telemedicine<br>Teleassistance	IS<br>Telecommunic	Management	This study shows how the iCOPE system promoted independent living and improves quality of life for

Year	Name of application	*Database*	*Objective*	*Type of group*	*Type of application*	*Technology applied*	*Domain of application*	*Significant Findings*
			elders with cognitive and functional impairment. This system allows: medication management, sleep activity monitoring, tele-physiotherapy and respiratory monitoring.			ation<br><br>Signal processing		elderly persons living alone. Also, can provide valuable backup since it operates round the clock.
2011	Support Environmen t [53]	Springer Link	Support Environment is a video monitoring system for caregivers to optimize their work and help them concentrate on their tasks at hand, reducing both mental and physical stress.	Primary caregivers	Telemedicine<br><br>Teleassistance	Telecommunic ation<br><br>IS	Management	The video monitoring system presented in this study, optimizes the work of the caregivers and helped the concentrate on their tasks at hand, reducing both mental and physical stresses. On the other hand, some caregivers expressed concerns over being watched by other caregivers through the monitor, especially when their activities were recorded.
2013	CaregiverN et [54]	Scopus	This system is a social support network of caregivers for locating and securing wandering patients, through a tracking device installed in a Subscriber Identity Module for Global System for Mobile Communications Network (GSM).	Informal caregivers	Mobile health<br><br>Teleassistance	Internet<br><br>Mobile telecommunic ations<br><br>Geolocation	Care<br><br>Quality of life	This study proposes a new type of intervention based on novel mobile application architecture to form and direct a social support network of caregivers for locating and securing wandering patients as soon as possible. However, the application has not yet been evaluated.
2013	Android-Based Mobile Application [55]	Google Scholar	The Android-based mobile application aims at assisting doctors in monitoring Alzheimer‘s patient medication.	Health professionals (Doctors)	Mobile health<br><br>Teleassistance	Internet<br><br>Mobile telecommunic ations	Care<br><br>Management	The response from the doctors shows that the proposed application is a suitable tool to support and assist doctors to monitor and communicate with their Alzheimer‘s patient. In future, further assessment

Year	Name of application	*Database*	*Objective*	*Type of group*	*Type of application*	*Technology applied*	*Domain of application*	*Significant Findings*
								need to be conducted to evaluate its usability among Alzheimer's patients and their caretakers.
2013	mobileWAY [56]	Scopus	The mobileWAY is a system that enables caregivers of persons with dementia (PwD) to remotely display dynamic, customized and illustrated information on the home television of the PwD.	Primary caregivers and informal caregivers	Mobile health<br><br>Teleassistance	Mobile telecommunic ations<br><br>Internet	Care<br><br>Quality of life	The results of the evaluations with mobileWAY seem to indicate that this system may offer potential for increasing self-steem and reducing behavioural disturbances of PwD with the use of photos and video for reminiscent therapy. One drawback of mobileWAY is the temporary or permanent attendance of the primary caregiver since it is neither replacing human caregiving nor a complete round-the-clock surveillance system.
2014	Understaid [57]	Google Scholar	UnderstAID is a platform that helps informal caregivers to understand and aid their relatives with dementia. It is an international project initiated by Denmark, Poland and Spain.	Informal caregivers	Teleassistance	Internet<br><br>Mobile telecommunci ations	Care<br><br>Quality of life	UnderstAID is a platform that helps informal caregivers to understand and aid their demented relatives. The platform is devised to be available in two versions, namely the light one for mobile appliance and the premium version. Also different activities leading to the popularization of the platform are planned. However, the study does not include evaluations of the platform with patients.
2014	Robot-ROSE [58]	Scopus	ROSE (Remotely Operated Service robot) was developed to perform home-care	Informal caregivers	Assistive technologies	Robotics<br><br>Aml	Care<br><br>Quality of	ROSE was developed to perform home-care tasks. Experiments with ROSE have shown this is feasible. The

Year	Name of application	*Database*	*Objective*	*Type of group*	*Type of application*	*Technology applied*	*Domain of application*	*Significant Findings*
			tasks. ROSE was controlled from a distance (8 km) by caregivers to perform small tasks for the elder. The design of ROSE can be used to develop service robots for other domains, e.g. to perform security tasks, building maintenance and construction.				life	design of ROSE can be used to develop service robots for other domains, e.g. to perform security tasks, building maintenance and construction

Abbreviations: IS: Information Systems, Aml: Ambience Intelligence
